# Impaired pulmonary function and associated factors in the elderly with tuberculosis on admission: a preliminary report

**DOI:** 10.1186/s12879-023-08183-2

**Published:** 2023-04-19

**Authors:** Meiyan He, Xiaoming Yang, Zunjing Zhang, Zhongda Liu

**Affiliations:** 1 Department of Tuberculosis, Lishui Hospital of Traditional Chinese Medicine, Zhejiang University of Traditional Chinese Medicine, No. 800 Zhongshan Road, Liandu District, Lishui, Zhejiang Province China; 2 Department of Respiratory Diseases, Lishui Hospital of Traditional Chinese Medicine Affiliated to Zhejiang University of Traditional Chinese Medicine, Lishui, 323000 China

**Keywords:** Elderly, Pulmonary tuberculosis, Pulmonary function, Risk factors, Severity

## Abstract

**Background:**

Pulmonary tuberculosis (TB) can impair pulmonary function (PF), especially in the elderly. The risk factors associated with the severity of PF impairment in the elderly with pulmonary TB remain unclear. Hence, this retrospective study aimed to address this issue to help improve the management of TB in the elderly population.

**Methods:**

From January 2019 to February 2022, the elderly who were admitted to our hospital for pulmonary TB and underwent PF testing were included in this analysis. The forced expiratory volume in one second percent of predicted (FEV1% predicted) and clinical characteristics were collected and analyzed retrospectively. The extent of impaired PF was then categorized based on the FEV1% predicted and classified as grade 1–5. Logistic regression analysis was used to analyze the risk factors for impaired PF.

**Results:**

A total of 249 patients who met the enrollment criteria were included in this analysis. According to the results of FEV1% predicted, all patients were classified as grade 1 (*n* = 37), grade 2 (*n* = 46), grade 3 (*n* = 55), grade 4 (*n* = 56), or grade 5 (*n* = 55). Statistical analysis showed that albumin (adjusted odds ratio (aOR) = 0.928, *P* = 0.013), body mass index (BMI) < 18.5 kg/m^2^ (aOR = 4.968, *P* = 0.046), lesion number ≥ 3 (aOR = 4.229, *P* < 0.001), male (aOR = 2.252, *P* = 0.009), respiratory disease (aOR = 1.669, *P* = 0.046), and cardiovascular disease (aOR = 2.489, *P* = 0.027) were related to the impairment of PF.

**Conclusions:**

PF impairment is common in the elderly with pulmonary TB. The male sex, BMI < 18.5 kg/m^2^, lesion number ≥ 3, hypoproteinemia, and respiratory and cardiovascular comorbidities were identified as risk factors for significant PF impairment. Our findings highlight the risk factors associated with PF impairment, which may be helpful to improve the current management of pulmonary TB in the elderly to save their lung function.

**Supplementary Information:**

The online version contains supplementary material available at 10.1186/s12879-023-08183-2.

## Background

Every year, approximately 10 million people fall ill with tuberculosis (TB), and 1.5 million people die from TB [1]. In the elderly, the TB epidemic is most prevalent in regions such as the Eastern Mediterranean, South-East Asia, and Western Pacific. In China, a total of 842,000 pulmonary TB patients were reported in 2021, and 206,000 of them were older than 65 years old; the incidence was estimated at 370/1,000,000, which is greater than that reported for other age groups [1]. Several factors coexist to make the management of elderly patients with TB a specific issue [2–4]. First, the elderly population has a poor tolerance to anti-TB therapy and their side effects are more frequent, making their treatment more complex. Second, the elderly have many more underlying diseases and age-related immunodepression, which increase the recurrence rate of disease and decrease the success rate of treatment. Third, the elderly with pulmonary TB usually have nonspecific symptoms, thus making an early diagnosis difficult. Besides, the elderly with TB diseases have a relatively higher mortality rate. Therefore, it is necessary to strengthen the management of TB in the elderly.

The association between pulmonary TB and pulmonary function (PF) impairment has been recognized [5, 6]. Pulmonary TB can lead to irreversible lung damage, which is visible as scarring, fibrosis, cavitation, or other types of damage revealed by radiological examinations [7]. Usually, patients have different levels of PF impairment, which can lead to a decreased quality of life, thus resulting in poor adherence to anti-TB therapy as well as disease relapse and recurrence [8]. Previously, most studies have focused on the impairment of PF after anti-TB therapy. However, the impairment of PF on admission is neglected and requires more investigation, especially for the elderly with pulmonary TB. Therefore, there is a need to evaluate the situation of chronic lung impairment among the elderly with TB in order to identify predictors of PF impairment and thus potential interventions that may be initiated emergently.

The main goal of this retrospective study was to detail the clinicopathological characteristics of the elderly with pulmonary TB and PF impairment, assess the severity of PF impairment, and estimate the risk factors associated with the severity of PF impairment on admission.

## Methods

### Ethics

This study protocol was approved by the Ethics Committee of the Lishui Hospital of Traditional Chinese Medicine (approval number: LW-016/2022). The requirement of informed consent was waived by the Clinical Ethics Committee of the Lishui Hospital of Traditional Chinese Medicine.

### Subjects

Between January 2019 and February 2022, the elderly with pulmonary TB were included for further analysis. The inclusion criteria were as follows: elderly patients (aged > 60 years) with a final diagnosis of pulmonary TB who underwent PF testing (without bronchodilator medication). The diagnosis of pulmonary TB was made based on the guidelines issued by the National Health and Family Planning Commission [9]. The exclusion criteria [10, 11] were as follows: (1) forced expiratory volume in one second percent of predicted (FEV1% predicted) ≥ lower limit of normal (LLN) [10]; (2) extrapulmonary TB; (3) mental disorders, epilepsy, impaired motor skills, severe liver or kidney dysfunction, or malignances; (4) myocardial infarction or shock during the past 3 months, unstable angina pectoris, uncontrolled hypertension (> 200 mm Hg systolic or > 100 mm Hg diastolic pressure), heart rate > 120 beats/min, or other acute pulmonary conditions (such as pneumothorax, massive hemorrhage, significant pleural effusion, acute exacerbation of chronic obstructive pulmonary disease (COPD)/asthma, and pulmonary embolism); (5) other contraindications to PF testing, such as aortic aneurysm or giant emphysematous bulla; (6) incomplete data (such as PF testing); (7) airway stenosis confirmed by chest computed tomography or bronchoscopy; (8) lung resection or abdominal surgery.

### PF

According to Chinese guidelines [10, 12], PF testing was performed in a room that was well ventilated and equipped with an air disinfection device and an ultraviolet lamp. In order to prevent TB from spreading during the PF test, the trained study investigator wore a gown, an N95 mask, a head cover, and gloves. During the inspection, each patient underwent PF examination separately and without contact with other patients. A disposable respiratory filter and a mouthpiece were used by the pulmonary TB patients, the inner tube and surface of the instrument were cleared with disinfectant, and the pulmonary function sensor and related parts were cleaned with multi-enzyme cleaner. Finally, an ultraviolet lamp and an air filter disinfector were used to disinfect the air. PF testing was performed by a trained study investigator using the MasterScreen PFT system (Jaeger, Germany), with the following parameters obtained: FVC% predicted, FEV1% predicted, and FEV1/FVC%. FEV1% predicted and FVC% predicted were standardized for age, sex, height, and weight. The impairment of PF was evaluated using FEV1% predicted. The features of ventilatory abnormality in spirometry include the following: restrictive, FVC% predicted < 80%; obstructive, FEV1/FVC < 0.92; and mixed, both restrictive and obstructive. The patients were classified as follows: grade 1, FEV1% predicted ≥ 70% and < LLN (or FEV1/FVC < LLN); grade 2, FEV1% predicted = 60–69%; grade 3, FEV1% predicted = 50–59%; grade 4, FEV1% predicted = 35–49%; grade 5, FEV1% predicted < 35% [10].

### Data collection

Data, such as sex, age, district, marital status, smoking habits, alcohol use, body mass index (BMI), disease duration (from symptom initiation to TB diagnosis) and underlying diseases (e.g., respiratory disease, cardiovascular disease, hypertension, and diabetes mellitus), were collected on admission. An active smoker was defined as someone who regularly smokes cigarettes (one cigarette per day, or consecutive usage for at least 1 year). An exsmoker was defined as someone who smoked more than 100 cigarettes in their lifetime but has not smoked recently. A nonsmoker was defined as someone who has never smoked or has smoked less than 100 cigarettes in their lifetime [13]. An acid-fast bacilli (AFB) smear, radiological examination (number of lesions and presence of cavitary lung lesions, assessed based on Chinese guidelines (No. WS 196–2001) [14], lab examinations (platelet, hemoglobin, and albumin), and PF examinations were performed within one week after admission, and the data were collected for further analysis.

### Statistical analysis

Data analysis was performed using SPSS 23.0. Quantitative data with a normal distribution are described as the mean ± standard deviation and were compared using the t-test; otherwise, the data are described as the median and interquartile range (IQR) and were compared using the Mann–Whitney or Kruskal–Wallis test. Categorical data are described as the count and percentage and were compared using the chi-squared test. The risk factors associated with PF impairment were analyzed using logistic regression analysis, and their corresponding odds ratios and 95% confidence intervals (CIs) were also calculated. A *P*-value less than 0.05 was considered significant.

## Results

### Patient characteristics

**Fig. 1:**
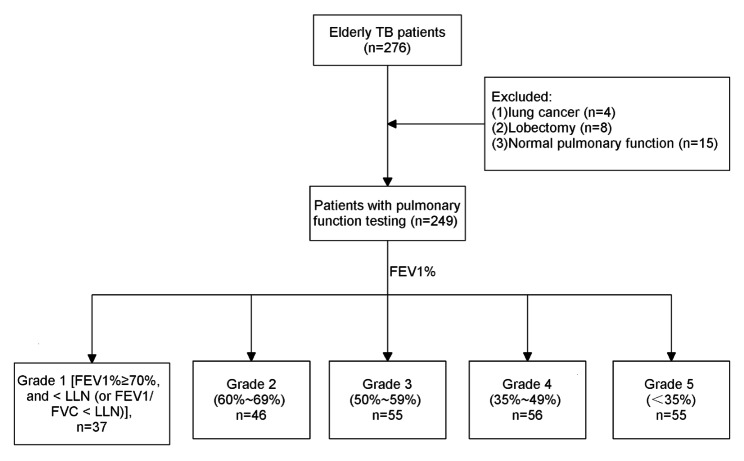
Flow chart of the patients included in this study

Table 1 shows the baseline characteristics of the included elderly patients with pulmonary TB. Twenty-seven elderly patients were excluded due to lung cancer (*n* = 4), lobectomy (*n* = 8), or normal pulmonary function (*n* = 15), and a total of 249 patients who met the enrollment criteria were included in the analysis (Fig. 1). Males accounted for 78.31% (*n* = 195) of all cases, and most of them were from rural areas (71.49%). Two hundred and twenty-one (88.76%) patients had initial treatment for pulmonary TB. Of these patients, 69.48% of them had a medical period of at least 1 year, 81.53% of them had at least three lesions, 47.79% of them were smear-positive, and 45.78% of them had lung diseases. In addition, their impaired PF was classified as obstructive (*n* = 151, 60.64%), restrictive (*n* = 17, 6.83%), or mixed obstructive-restrictive (*n* = 81, 32.53%).

**Table 1:** The clinical characteristics of the included elderly patients with pulmonary tuberculosis (TB).

Variable	Number (%)
**Sex**	
Male	195 (78.31)
Female	54 (21.69)
**Age, years**	65 (60–69)
**Height, cm**	163 (158–168)
**Weight, kg**	54 (48–61)
**Body mass index, kg/m** ^**2**^	20.29 (18.22–22.81)
**Smoking**	129 (51.81)
**Alcohol use**	73 (29.32)
**Marital status**	
Unmarried	5 (2.01)
Married	212 (85.14)
Divorced or widowed	32 (12.85)
**District**	
Rural	178 (71.49)
County	71 (28.51)
**Underlying disease**	
Respiratory disease	114 (45.78)
Cardiovascular disease	27 (10.84)
Hypertension	48 (19.28)
Diabetes mellitus	55 (22.09)
**Disease duration (years)**	
<1	173 (69.48)
1–3	26 (10.44)
≥3	50 (20.08)
**Tuberculosis**	
Initial treatment for TB	221 (88.76)
Retreatment for TB	19 (7.63)
Drug-resistant TB	9 (3.61)
**Cavitary lung lesions**	62 (24.90)
**Lesion number ≥ 3**	203 (81.53)
**AFB smear (+)**	119 (47.79)
**Albumin (g/L)**	34.67 ± 4.85
**Hemoglobin (g/L)**	125.65 ± 17.30
**Platelet (10** ^**9**^ **/L)**	239.00 ± 92.28
**Ventilatory dysfunction**	
Obstructive	151 (60.64)
Restrictive	17 (6.83)
Mixed	81 (32.53)
**FEV1, % predicted**	50.76 ± 18.24
**FVC, % predicted**	57.06 ± 20.72
**FEV1/FVC, %**	84.39 ± 16.84

FEV1, forced expiratory volume in one second; FVC, forced vital capacity

The mean ± standard deviation values of FEV1% predicted, FVC% predicted, and FEV1/FVC% were 50.76 ± 18.24%, 57.06 ± 20.72%, and 84.39 ± 16.84%; the FEV1/FVC ratios were 81.04 ± 16.58 for patients with < 3 lesions and 85.14 ± 16.84 for ≥ 3 lesions, and 85.51 ± 13.14 for patients with smear (-) and 83.20 ± 20.10 for smear (+). The FEV1(%) was 51.11 ± 17.15 for patients with < 3 lesions and 50.66 ± 18.01 for ≥ 3 lesions, and 54.48 ± 17.48 for patients with smear (-) and 47.06 ± 17.99 for smear (+) (see Table S1 in the Supplementary materials for more details). According to the FEV1% predicted results, the patients were classified as grade 1 (*n* = 37), grade 2 (*n* = 46), grade 3 (*n* = 55), grade 4 (*n* = 56), or grade 5 (*n* = 55).

### Comparisons among the groups of different grades

Table 2 shows the comparison of the clinical characteristics between the groups of different grades. Statistical analysis showed that significant differences were observed in terms of age, sex, marital status, BMI, disease duration, respiratory disease, cardiovascular disease, lesions, albumin, and hemoglobin among the groups of different grades (all *P* < 0.05). However, no significant differences were observed for the other variables, such as smoking habits, alcohol use, district, diabetes mellitus, hypertension, cavitary lung lesions, AFB smear, and platelets among the groups of different grades (all *P* > 0.05).

**Table 2:** Comparison of the clinical characteristics of patients with pulmonary TB.

Variable		Grade 1 (*n* = 37, %)	Grade 2 (*n* = 46, %)	Grade 3 (*n* = 55, %)	Grade 4 (*n* = 56, %)	Grade 5 (*n* = 55, %)	*χ*^*2*^/*Z*	*P*
Sex	Male	23 (62.16)	33 (71.34)	43 (78.18)	48 (85.71)	47 (85.45)	8.980	0.003
	Female	14 (37.84)	13 (28.66)	12 (21.82)	8 (14.29)	8 (14.55)		
Age, years	60–69	30 (81.08)	43 (93.48)	41 (74.54)	43 (76.79)	29 (52.73)	19.431	< 0.001
	70–79	7 (18.92)	3 (6.52)	12 (21.82)	11 (19.64)	19 (34.54)		
	80–90	0 (0.00)	0 (0.00)	2 (3.64)	2 (3.57)	7 (12.73)		
Smoking	Yes	17 (45.95)	21 (45.65)	31 (56.36)	35 (62.50)	25 (45.45)	0.267	0.606
	No	20 (54.05)	25 (54.35)	24 (43.64)	21 (37.50)	30 (54.55)		
Alcohol use	Yes	9 (24.32)	10 (21.74)	18 (32.73)	21 (37.50)	15 (27.27)	0.882	0.348
	No	28 (75.68)	36 (78.26)	37 (67.27)	35 (62.50)	40 (72.73)		
Marital status	Unmarried	1 (2.70)	2 (4.35)	1 (1.82)	0 (0.00)	1 (1.82)	6.744	0.034
	Married	32 (86.49)	42 (91.30)	47 (85.45)	51 (91.07)	40 (72.73)		
	Divorced or widowed	4 (10.81)	2 (4.35)	7 (12.73)	5 (8.93)	14 (25.45)		
District	Rural	23 (62.16)	34 (73.91)	40 (72.73)	36 (64.29)	45 (81.82)	1.770	0.183
	County	14 (37.84)	12 (26.09)	15 (27.27)	20 (35.71)	10 (18.18)		
BMI (kg/m^2^)	< 18.5	6 (16.22)	6 (13.04)	10 (18.18)	17 (30.36)	29 (52.73)	23.507	< 0.001
	18.5–24	26 (70.27)	29 (63.04)	35 (63.63)	33 (58.93)	22 (40.00)		
	24–28	4 (10.81)	9 (19.57)	8 (14.55)	5 (8.93)	4 (7.27)		
	≥ 28	1 (2.70)	2 (4.35)	2 (3.64)	1 (1.78)	0 (0.00)		
Disease duration (years)	< 1	33 (89.19)	36 (78.26)	41 (74.55)	38 (67.86)	25 (45.45)	21.871	< 0.001
	1–3	0 (0.00)	6 (13.04)	3 (5.45)	6 (10.71)	11 (20.00)		
	≥ 3	4 (10.81)	4 (8.70)	11 (20.00)	12 (21.43)	19 (34.55)		
Respiratory disease^a^	Yes	9 (24.32)	17 (36.96)	27 (49.09)	27 (48.21)	36 (65.45)	15.499	< 0.001
	No	28 (75.68)	29 (63.04)	28 (50.91)	29 (51.79)	19 (34.55)		
Cardiovascular disease^b^	Yes	3 (8.11)	1 (2.17)	5 (9.09)	4 (7.14)	14 (25.45)	9.602	0.002
	No	34 (91.89)	45 (97.83)	50 (90.91)	52 (92.86)	41 (74.55)		
Hypertension	Yes	7 (18.92)	10 (21.74)	8 (14.55)	13 (23.21)	11 (20.00)	0.059	0.808
	No	30 (81.08)	36 (78.26)	47 (85.45)	43 (76.79)	44 (80.00)		
Diabetes mellitus	Yes	12 (32.43)	10 (21.74)	13 (23.64)	13 (23.21)	9 (16.36)	2.159	0.142
	No	25 (67.57)	36 (78.26)	42 (76.36)	43 (76.79)	46 (83.64)		
Cavitary lung lesions	Yes	9 (24.32)	10 (21.74)	11 (20.00)	15 (26.79)	17 (30.91)	1.162	0.281
	No	28 (75.68)	36 (78.26)	44 (80.00)	41 (73.21)	38 (69.09)		
Lesions ≥ 3	Yes	18 (48.65)	30 (65.22)	50 (90.91)	53 (94.64)	52 (94.55)	39.150	< 0.001
	No	19 (51.35)	16 (34.78)	5 (9.09)	3 (5.36)	3 (5.45)		
AFB smear	(-)	23 (62.16)	20 (43.48)	28 (50.91)	32 (57.14)	27 (49.09)	0.097	0.755
	(+)	14 (37.84)	26 (56.52)	27 (49.09)	24 (42.86)	28 (50.91)		
Albumin (g/L)		38.11 ± 4.38	35.87 ± 4.31	34.00 ± 4.85	34.22 ± 4.48	32.47 ± 4.57	9.785	< 0.001
Hemoglobin (g/L)		131.95 ± 15.49	128.41 ± 14.47	125.04 ± 16.16	124.13 ± 16.75	121.29 ± 20.93	9.785	< 0.001
Platelet (10^9^/L)		237.97 ± 91.55	236.24 ± 90.24	236.78 ± 95.29	247.38 ± 101.35	235.677 ± 84.23	0.150	0.963

BMI, body mass index; AFB, acid-fast bacilli; ^a^ Chronic obstructive pulmonary disease; asthma (after remission)

^b^ Cardiovascular diseases include stable coronary heart disease and hyperlipidemia

### Multivariate logistic regression analysis

Further multivariate logistic regression analysis was performed to identify the risk factors associated with PF impairment. Variables with *P*-values less than 0.05 in univariate analysis were selected for multivariate analysis. Finally, six variables (male sex, BMI < 18.5 kg/m^2^, lesion number ≥ 3, hypoproteinemia, and respiratory and cardiovascular comorbidities) were left in the model (all *P* < 0.05). In addition, the parallel line test showed a *P*-value of 0.819, indicating that the regression equations are parallel to each other. The statistical analysis showed that albumin (adjusted odds ratio (aOR) = 0.928, *P* = 0.013), BMI < 18.5 kg/m^2^ (aOR = 4.968, *P* = 0.046), lesion number ≥ 3 (aOR = 4.229, *P* < 0.001), male (aOR = 2.252, *P* = 0.009), respiratory disease (aOR = 1.669, *P* = 0.046), and cardiovascular disease (aOR = 2.489, *P* = 0.027) were all related to the impairment of PF (Table 3). Furthermore, albumin and BMI were negatively associated with the risk of impaired PF; while lesion number ≥ 3, cardiovascular disease, male, and respiratory disease increased the risk of impaired PF.

**Table 3:** Multivariate logistic regression analysis of the association between variables in patients with pulmonary TB.

Variable		Regression coefficient	Standard error	*P*	aOR	95% CI		
**Grade**	1	-4.239	1.663	6.499				
	2	-2.817	1.66	2.88				
	3	-1.56	1.656	0.888				
	4	-0.154	1.648	0.009				
**Albumin (g/L)**		-0.075	0.03	0.013	0.928	0.875,0.984		
**Hemoglobin (g/L)**		-0.008	0.008	0.369	0.992	0.976, 1.009		
**BMI (kg/m** ^**2**^ **)**	< 18.5	1.603	0.804	0.046	4.968	1.028, 23.999		
	18.5–24	0.575	0.772	0.456	1.777	0.391, 8.069		
	24–28	1.139	0.824	0.167	3.124	0.621,15.721		
	≥ 28	–	–	–	1.000	–		
**Disease duration (years)**	< 1	-0.548	0.327	0.094	0.578	0.305, 1.099		
	1–3	0.228	0.483	0.637	1.256	0.487, 3.238		
	≥ 3	–	–	–	1.000	–		
**Respiratory disease**								
Yes		0.512	0.256	0.046	1.669	1.010, 2.759		
No		–	–	–	1.000	–		
**Lesions (≥ 3)**								
Yes		1.442	0.345	< 0.001	4.229	2.149, 8.314		
No		–	–	–	1.000	–		
**Age (years)**	60–69	-1.225	0.684	0.073	0.294	0.077, 1.122		
	70–79	-0.734	0.709	0.300	0.480	0.119, 1.927		
	80–90	–	–	–	1.000	–		
**Cardiovascular disease**								
Yes		0.912	0.411	0.027	2.489	1.112, 5.573		
No		–	–	–	1.000	–		
**Male**		0.812	0.312	0.009	2.252	1.221, 4.158		

aOR, adjusted odds ratio; CI, confidence interval; BMI, body mass index

## Discussion

The impairment of PF is common in patients with pulmonary TB. However, the extent of impaired PF in the elderly with pulmonary TB remains unclear. This study quantitatively evaluated the extent of PF impairment in elderly patients with TB before anti-TB therapy by measuring the FEV1% predicted value. In addition, the risk factors associated with the severity of PF impairment, which may be helpful to improve the management of pulmonary TB among the elderly, were investigated. The findings demonstrated that pulmonary TB causes significant impairment of PF and that albumin, BMI < 18.5 kg/m^2^, lesion number ≥ 3, cardiovascular disease, male, and respiratory disease were associated with impairment of PF.

Our data suggest that, when treating the elderly with pulmonary TB complicated with impaired PF, besides anti-TB therapy, attention should be paid to the nutritional status of the patients and therapy should be administered in a timely fashion. For example, the BMI and protein intake should be monitored. In addition, the management of underlying diseases (such as cardiovascular and respiratory diseases) is also a key factor to improve the PF of these elderly patients.

Low albumin and BMI values were identified as risk factors for impairment of PF. This may be explained by the association between malnutrition and impairment of PF. Malnutrition is one of the most important risk factors associated with TB development. An estimated 2.3 million TB cases have been attributed to malnutrition, which is more common than other causes (such as human immunodeficiency virus and diabetes mellitus) [15]. Malnutrition may result from insufficient energy intake [16], which presents with changes in the body composition, metabolism, and immune status. Furthermore, impairment of PF may then occur due to reduced physical activity (muscle atrophy) [17, 18]. The associations of albumin and BMI with PF have been addressed in several studies [19]. For example, BMI and albumin have been reported to be independently associated with FEV1% predicted in children with cystic fibrosis [19]. In addition, a correlation between FEV1 and BMI has been found by Popova et al. in patients treated for pulmonary TB, with a level of 0.14 (*P* < 0.05) [20]. Moreover, Khatri et al. have shown that albumin levels are positively correlated with FEV1% predicted among patients with asthma (R = 0.378; *P* = 0.010) [21].

In addition, inconsistent trends of BMI (≥ 28, 24–28, 18.5–24, and < 18.5) and disease duration (< 1, 1–3, and ≥ 3) were observed. The inconsistence of BMI may be explained by the following factors. First, the normal population has a BMI of 18.5–24, which has no significant impact on the pulmonary function. Second, those who are overweight or obese have a close association with the PF [22, 23]. Third, our findings demonstrated that when the BMI was less than 18.5, a positive correlation with PF was observed for the BMI. Thus, this reverse relationship between overweight or underweight and PF may be the main reason for the inconsistency. A similar explanation contributes to the disease duration. We found that when the duration was longer than 1 year, there was more severe PF impairment. However, when the duration was shorter than 1 year, there was much less PF impairment.

A lesion number ≥ 3 was associated with a decreased FEV1. This finding suggests that impairment of PF is more likely to occur in the elderly TB patients with more lesions. Similarly, in a previous study, multilobar involvement has been associated with a marked lung function decline [24]. A report by Long et al. [25], who described that functional impairment is proportional to the number of diseased segments, is also consistent with our findings. This may be explained by three key factors: (1) immunosenescence, various anatomical and physiological changes linked to ageing, as well as malnutrition and comorbidities in the elderly can lead to the increased susceptibility of lung infection [2]; (2) the immunocompromised status resulting from underlying diseases (e.g., steroid use, immunosuppressant use, etc.) makes the situation more complex [26, 27]; and (3) pulmonary TB can lead to irreversible lung parenchymal destruction and lung remodeling [25, 28]. Therefore, in the elderly with pulmonary TB, chest computed tomography features include parenchymal density, cavitary lung lesions, ground glass opacities, and miliary nodules [29]. In addition, more segments distributed with lesions have been found in the elderly with pulmonary TB compared to those with community-acquired pneumonia [30].

Respiratory disease was also demonstrated to be a risk factor for impaired PF. The findings from several studies are consistent with our report. For example, Lee et al. have found that the FVC and postbronchodilator FEV1 of patients with chronic airflow obstruction were less than those of COPD patients (*P* < 0.05) [31]. Furthermore, Yang et al. have determined that severe obstructive ventilatory disorders are associated with more respiratory symptoms, and among subjects with prior TB, 29% developed obstructive ventilatory disorders [32]. Abnormal lung function associated with pulmonary TB was also observed in 18–94% of patients. Additionally, Byrne et al. have stated that the presence of other respiratory comorbidities is one of the risk factors associated with the extent of the lung function abnormality [33]. In a previous study, 84% of patients with respiratory complaints had either obstructive or restrictive patterns on spirometry. However, all asymptomatic patients had normal spirometry readings [34]. Moreover, Poh et al. have reported that patients with respiratory symptoms have a higher risk of developing obstructive airway disease than asymptomatic patients [35]. These findings demonstrate that smoking, a married status, and BMI (24–28 kg/m^2^) were associated with obstructive ventilatory disorders and that age (70–79 years) and disease duration (1–3 years) were associated with restrictive ventilatory disorders (see Tables S2–S3 in the Supplementary materials for more information).

Cardiovascular disease was another risk factor for impaired PF that was identified in this study. PF is known to be related to the incidence of cardiovascular disease [36]. Although the reasons for their association remain unclear, indirect evidence has been found in several studies. For example, COPD patients frequently have cardiac disease [37]. In addition, an elevated B-type natriuretic peptide level has been observed in COPD patients [38]. FEV1 and FVC also have been associated with cardiac parameters [39, 40]. Remarkably, FEV1 and FVC by spirometry are associated with smaller ventricular volumes and a reduced ventricular mass as determined by cardiovascular magnetic resonance imaging [41].

Finally, the male sex was found to be a risk factor for impaired PF among the elderly TB patients. This may be explained by the fact that men have longer airways than women, causing greater specific resistance in the respiratory tract [42]. Our findings are consistent with a previous report by Berglund et al. [43].

There are several limitations of this study that must be addressed. First, this study had a retrospective nature. Second, this study was conducted at a single center. Therefore, selection bias might have influenced the findings. Third, PF testing was performed at the time of admission. The anti-TB therapy could have partially improved the PF at the end of treatment. The comparison of PF between before and after treatment might be useful to understand its clinical significance among the elderly with pulmonary TB. Therefore, further evidence is required to validate our findings.

## Conclusions

In conclusion, PF impairment is common in the elderly with pulmonary TB. Male patients as well as those with BMI < 18.5 kg/m^2^, lesion number ≥ 3, hypoproteinemia, or respiratory or cardiovascular comorbidities had a higher risk of experiencing significant PF impairment, compared with the others. Our findings strengthen the need for an early diagnosis of pulmonary TB in the elderly and will be helpful to improve the current management of pulmonary TB among the elderly to save their lung function.

## Electronic supplementary material

Below is the link to the electronic supplementary material.

Supplementary materials: Table S1. The FEV1/FVC ratio and FEV1(%) between different groups of patients. Table S2. Logistic regression analysis of obstructive ventilatory disorders. Table S3 Logistic regression analysis of restrictive ventilatory disorders.

## Data Availability

The datasets used and/or analyzed during the current study are available from the corresponding author on reasonable request.

## List of abbreviations

TB: tuberculosis
PF: pulmonary function
FEV1: The forced expiratory volume in one second
COPD: chronic obstructive pulmonary disease
FVC: forced vital capacity
LIN: lower limit of normal
BMI: body mass index
AFB: acid-fast bacilli
IQR: interquartile range
CIs: confidence intervals.
