# Supplementary material for: Impaired pulmonary function and associated factors in the elderly with tuberculosis on admission: a preliminary report
Source: BMC Infect Dis. 2023 Apr 19;23:251. doi: 10.1186/s12879-023-08183-2 (PMC10116730; doi:10.1186/s12879-023-08183-2)
Supplement: Supplementary file 1 — Supplementary materials: Table S1. The FEV1/FVC ratio and FEV1(%) between different groups of patients. Table S2. Logistic regression analysis of obstructive ventilatory disorders. Table S3 Logistic regression analysis of restrictive ventilatory disorders. [file 12879_2023_8183_MOESM1_ESM.pdf]

Supplementary materials:

Table S1. The FEV1/FVC ratio and FEV1(%) between different groups of patients.

	FEV1/FVC, %	FEV1, % predicted
Lesion number<3	81.04±16.58	51.11±17.15
Lesion number≥3	85.14±16.84	50.66±18.01
Acid-fast bacilli smear (-)	85.51±13.14	54.48±17.48
Acid-fast bacilli smear (+)	83.20±20.10	47.06±17.99

Table S2. Logistic regression analysis of obstructive ventilatory disorders.

Variable		aOR	95%CI	*P*
Smoking	Yes	2.845	1.621,4.993	<0.001
	No	1	–	–
Marital status	Unmarried	0.112	0.010,1.246	0.075
	Married	0.359	0.159,0.810	0.014
	Divorced or widowed	1	–	–
BMI (kg/m^2)	<18.5	0.343	0.056,2.110	0.248
	18.5–24	0.192	0.032,1.148	0.070
	24–28	0.094	0.013,0.676	0.019
	≥28	1	–	–

aOR, adjusted odds ratio; CI, confidence interval; BMI, body mass index.

Table S3 Logistic regression analysis of restrictive ventilatory disorders.

Variable		aOR	95%CI	*P*
	60–69	1	–	–
Age (years)	70–79	0.343	0.159,0.741	0.006
	80–90	0.643	0.076,5.428	0.685
Disease	<1	1.388	0.392,4.910	0.611
duration	1–3	0.245	0.106,0.567	0.001
(years)	≥3	1	–	–

aOR, adjusted odds ratio; CI, confidence interval.
